# Dynamic parameter estimation and prediction over consecutive scales, based on moving horizon estimation: applied to an industrial cell culture seed train

**DOI:** 10.1007/s00449-020-02488-1

**Published:** 2020-12-29

**Authors:** Tanja Hernández Rodríguez, Christoph Posch, Ralf Pörtner, Björn Frahm

**Affiliations:** 1 Ostwestfalen-Lippe University of Applied Sciences and Arts, Biotechnology and Bioprocess Engineering, Lemgo, Germany; 2 Novartis Technical Research and Development, Sandoz GmbH, Langkampfen, Austria; 3 Institute of Bioprocess and Biosystems Engineering, Hamburg University of Technology, Hamburg, Germany

**Keywords:** Dynamic parameter estimation, Bioprocess, Cell cultures, Moving horizon estimation, Prior knowledge

## Abstract

Bioprocess modeling has become a useful tool for prediction of the process future with the aim to deduce operating decisions (e.g. transfer or feeds). Due to variabilities, which often occur between and within batches, updating (re-estimation) of model parameters is required at certain time intervals (dynamic parameter estimation) to obtain reliable predictions. This can be challenging in the presence of low sampling frequencies (e.g. every 24 h), different consecutive scales and large measurement errors, as in the case of cell culture seed trains. This contribution presents an iterative learning workflow which generates and incorporates knowledge concerning cell growth during the process by using a moving horizon estimation (MHE) approach for updating of model parameters. This estimation technique is compared to a classical weighted least squares estimation (WLSE) approach in the context of model updating over three consecutive cultivation scales (40–2160 L) of an industrial cell culture seed train. Both techniques were investigated regarding robustness concerning the aforementioned challenges and the required amount of experimental data (estimation horizon). It is shown how the proposed MHE can deal with the aforementioned difficulties by the integration of prior knowledge, even if only data at two sampling points are available, outperforming the classical WLSE approach. This workflow allows to adequately integrate current process behavior into the model and can therefore be a suitable component of a digital twin.

## Introduction

Mathematical models are playing an important role in simulation and prediction of bioprocesses. Model-based methods are applied in the context of model-assisted Design of Experiments, the design, layout and optimization of production processes [19, 20, 28, 31, 32, 36, 38, 44]. Furthermore, they are used as predictive models, enabling prediction of the process future, featuring the development of decision making, optimization and control strategies [9, 11, 29, 36]. The performance mainly depends on prediction accuracy of the model which in turn depends on data quality [39], the complexity of the model and the ability to address batch-to-batch variabilities [43]. The latter can occur concerning cell growth, viability as well as uptake and production rate. They represent a challenging task, especially in the case of mammalian cell cultures. These are the most frequently used hosts for many biopharmaceuticals (e.g. antibodies and proteins for diagnostic and therapeutic purposes) [42].

**Fig. 1:**
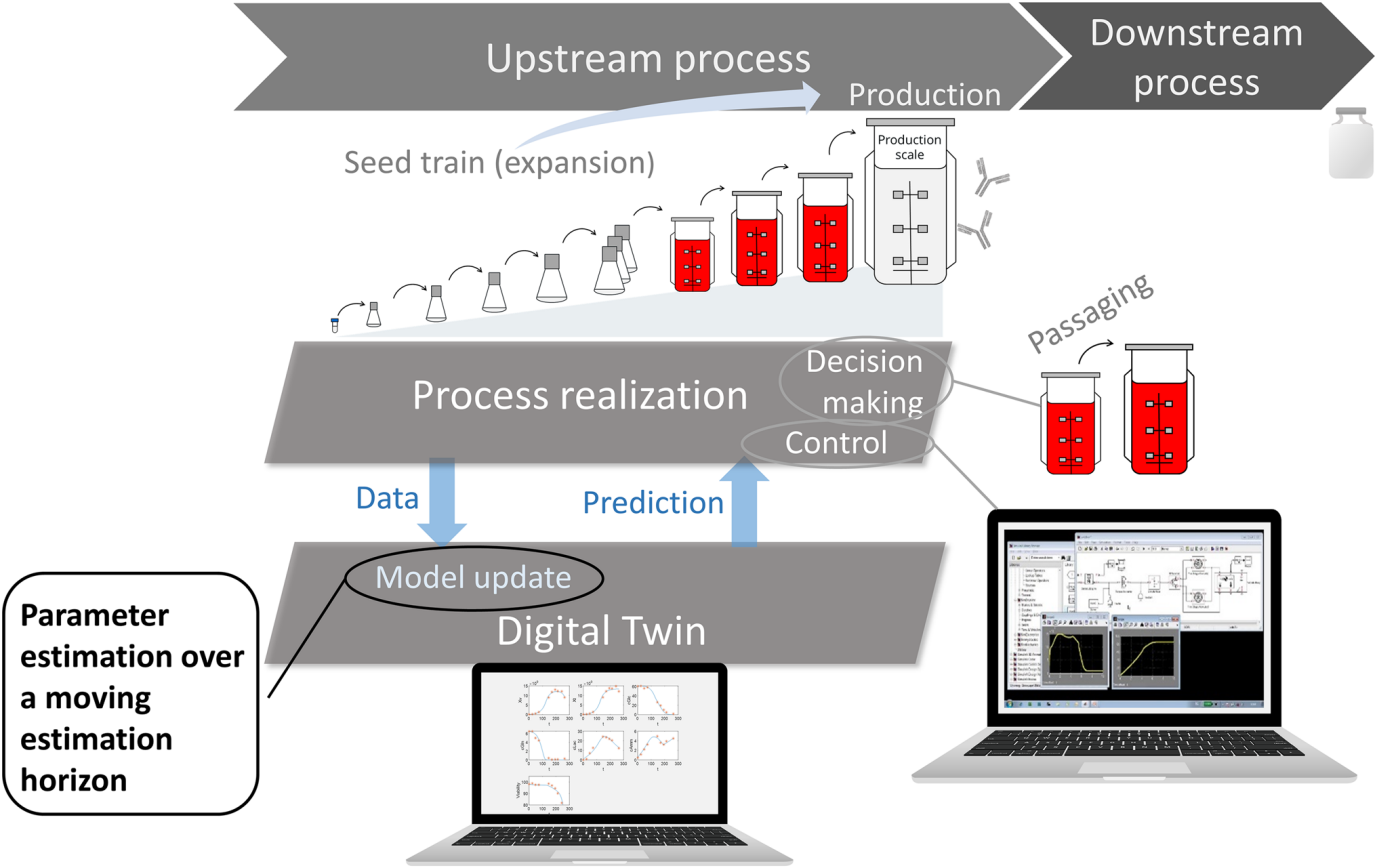
Role of parameter estimation techniques within a biopharmaceutical production process

An iterative model updating procedure, meaning that model parameters are updated (re-estimated) after certain time steps, is required to take possible variabilities into account. Figure 1 illustrates the role of this model updating procedure within the production process steps. Data from the realized ongoing process was used to generate a digital twin of the process, that means a virtual representation of the process which provides a prediction of the remaining future. This prediction is then used to deduce operating decisions, e.g. concerning cell passaging [8, 14, 18] or to control the process, e.g. through an Open-Loop-Optimal-Control (OLFO) method for the control of optimal feeding [11, 26] or to control pH and temperature shifts [35]. As soon as new process data become available model updating, meaning re-estimation of model parameters over a growing estimation horizon (window), is performed (= dynamic parameter estimation).

In literature, different approaches for re-estimation of model parameters over a growing database can be found [10, 12, 13, 17]. They differ in terms of the objective function which has to be optimized, and in terms of the considered estimation horizon (number of data points used for parameter estimation). This can be growing or moving with a fixed horizon length. Furthermore, re-estimation approaches differ in terms of the applied optimization algorithm (e.g. Nelder-Mead Simplex optimization [34] or particle swarm optimization [45]). Besides, estimation techniques can be divided into frequentist approaches (searching a point estimate, i.e. one single value for each parameter) and probabilistic approaches (searching an interval estimate, based on a probability distribution for each parameter). While frequentist parameter estimation can be combined with uncertainty analysis, probabilistic parameter estimation already considers uncertainty quantification within the estimation procedure. One probabilistic approach is the Bayesian parameter estimation. This approach includes uncertainty quantification and consideration of prior knowledge concerning model parameters. This prior knowledge is quantified in form of probability distributions. As soon as new data points are added, these distributions are updated. Some examples within the field of biochemical engineering can be found in literature [15, 22, 44]. However, frequentist approaches are the predominating estimation techniques and therefore considered in this study. Two estimation techniques are applied and their robustness and impact on prediction performance are investigated, a classical weighted least squares estimation (WLSE) approach and a moving horizon estimation (MHE) technique. While the objective function of the WLSE only considers information from data points within the estimation horizon, the objective function of the MHE approach includes a term containing prior knowledge concerning the parameter values and a penalty term for the change in parameter values [10, 12, 37].

While typical applications found in literature cover cultivations in one scale [10, 17], no recommendations can be found for re-estimation and prediction of cultivations over several consecutive scales as in the case of cell culture seed trains. However, these are required for the production of biopharmaceuticals in suspension culture to increase the cell number from cell thawing up to production scale [8, 18]. Cells are cultivated in cultivation systems of different volumes starting with small volumes (typically with shake flasks) and they are passaged every 3–4 days into the next bigger one (using bioreactors in larger scales). It has been shown that cultivation conditions during the seed train have a significant impact on cell performance in production scale [21]. Consequently, simplifying or enabling better decision making for seed train operations is required. Moreover, monitoring, control and development of optimization strategies play an important role.

Nevertheless, predictive modeling in case of only a few available data points are rarely considered in the literature. For various fields, such as many microbial and yeast processes, a relatively high density of data points is available, typically from on-line measurements and in combination with soft sensors, whereas cell culture processes are usually characterized by a low data density and samples are often taken only once or twice a day. In this contribution, it is investigated how to integrate current knowledge into dynamic optimization to address possible variabilities, especially focusing on the impact of the estimation horizon (data points used for parameter estimation) and the objective function on model parameters and prediction performance. It is shown how changes in cell growth behavior can be detected through changes in model parameter values after updating model parameters. A workflow, containing the recommended steps was developed and applied to an industrial CHO cell culture seed train. Finally, similarities between the Moving Horizon Estimation technique and the Bayesian approach are described.

## Materials and methods

In this study, different scenarios concerning estimation horizon and updating strategy are considered. Without loss of generality, they are explained for application to cultivation data over several consecutive scales (a seed train) and assuming that data from several consecutive seed trains are collected. Furthermore, two different estimation techniques having different objective functions [weighted least squares estimation (WLSE) and moving horizon estimation (MHE)], were applied.

### Estimation and prediction horizon

Re-estimation of model parameters (or model updating) is performed at different points in time during the process. Therefore, the time span $t_1,...,t_{\rm end}$ of the process (here explained for a seed train) is divided at the current point in time $t_i$ into an estimation horizon of length $n, \, t_{i-n},...,t_{i}$, containing the *n* most recent data points, and the prediction horizon, $t_{i+1},...,t_{\rm end}$.

During prediction of one seed train, the right bound (the end) of the prediction horizon is kept fixed while the left bound (the beginning) of the prediction horizon moves forward with new measurements (receding prediction horizon).

**Fig. 2:**
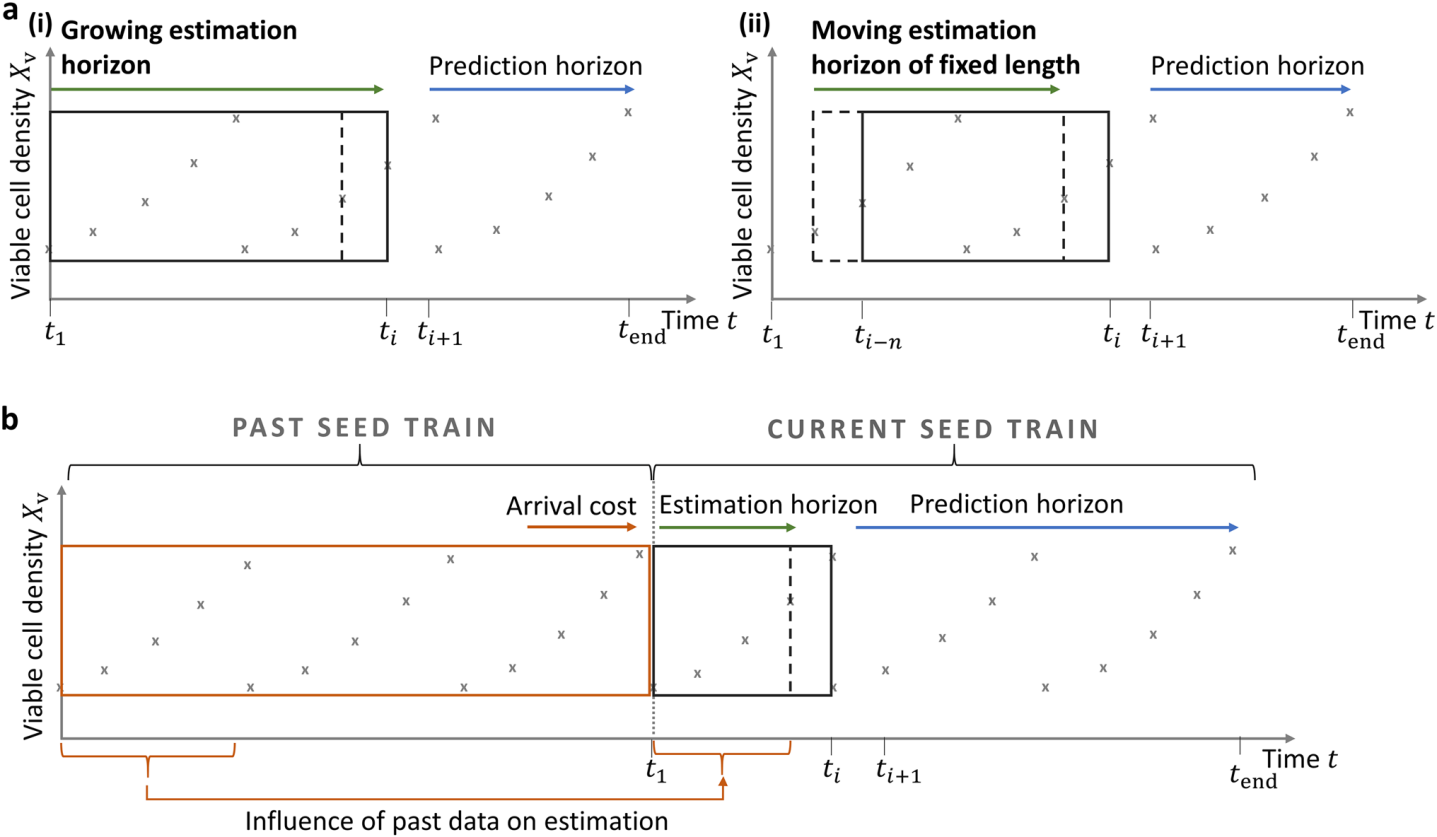
Scheme of different estimation horizons. **a** (i) Growing estimation horizon and (ii) moving estimation horizon of fixed length, both applied within the weighted least squares estimation (WLSE) technique; **b** Growing estimation horizon including an arrival cost window, applied within the moving horizon estimation (MHE) technique

At the same time, the right bound of the estimation horizon moves forward (*moving estimation horizon*). For the left bound of the estimation horizon different scenarios are applied (see Fig. 2a): (i) Fixed point in time at the beginning of the current seed train or at the beginning of each cultivation scale (described in this work as *growing estimation horizon*), (ii) moving left bound, meaning that as soon as a new data point becomes available, the oldest data point is excluded from the estimation horizon (described in this work as *moving estimation horizon of fixed length*).

### Objective functions

The two applied objective functions (estimation techniques) in this study are presented below. To avoid confusion concerning the terminologies containing ’moving horizon’ it should be noted, that the ’*moving estimation horizon*’ as described above refers to the data used directly within parameter estimation, whereby ’*moving horizon estimation*’ refers to an estimation technique characterized through a specific objective function which will be explained below.

*Weighted Least Squares Estimation (WLSE)* Weighted non-linear least square methods are widely applied estimation methods for static optimization problems [6, 13]. The aim is to minimize the squared deviation between *n* measured and simulated data points for *k* variables, $y_{m,j}=(y_{m,j1},...,y_{m,jn})$ and $y_j=(y_{j1},...,y_{jn})$, respectively, for a variable $y_j$, multiplied by a weighting factor $w_{m,ij}$ for $i=1,...,n$ and $j=1,...,k$ (e.g. to address measurement deviations and to compare quantities of different dimensions or assigning higher importance to specific quantities which is often done because some measured values or data points might be more reliable compared to others) for a fixed period of time $t=(t_1,...,t_n)$. Here, the weighting factor for variable $y_j$ was determined through division by the maximum experimental value $y_{j,\rm max}$ over time span *t*, thus $w_{m,ij} = \frac{1}{y_{j,\rm max}}$. This can be formulated as an static optimization problem, consisting in minimizing the following objective function *J* over the space of model parameters $p=(p_1,...,p_{n_p})$:

1 $$\begin{aligned} J(p) = \sum _{j=1}^{k} \sum _{i=1}^{n} w_{m,ij} \cdot (y_{m,ij} - {y_{ij}(p)})^2 \end{aligned}$$

This notation is similar to $J = (y_m - y)^T W_m (y_m - y)$, also found in literature. This estimation method is simple to apply but suffers from sensitivities to outliers and high measurement deviations, when applied during dynamic optimization [13].

*Moving horizon estimation (MHE)* Now, if more and more data are collected (e.g. from several seed trains) and past and present data are used for parameter estimation (dynamic parameter estimation), the computational cost of a simple least square estimation increases. As stated in [10], an adequate approach to prevent computational limitations is to formulate the parameter estimation over a fixed size estimation horizon and to include information obtained from past data through a so called *arrival cost term* (sometimes called *forgetting term*). This method, presented in [37], is known as a moving horizon estimation (MHE) and is divided into two main parts, state estimation and arrival cost estimation. A fixed estimation horizon is defined containing measurements used for the state estimation. Every time new measurements are supplied, old measurements are discarded and passed from the estimation horizon to the so called *arrival cost window* (or historical window), whenever they exceed a fixed estimation horizon length. Information from the past (up to the estimation horizon) is now summarized within the arrival cost term and through a penalty term for the change in parameters. Figure 2b illustrates how cultivation data from various seed trains can be divided iteratively into an arrival cost window, an estimation horizon and a prediction horizon. Different approaches to estimate the arrival cost can be found in the literature [10, 12, 13, 41].

In this contribution, a *squared-error moving horizon estimation (MHE)* was applied. The aim is to add penalty terms to the weighted squared errors (see eq. 1), taking into account learned information from historical batches (cultivations) of a fixed arrival cost window size (e.g. one preceding seed train). One term contains the prior values $\hat{y_j}=(\hat{y}_{j1},...\hat{y}_{jn})$ resulting from simulations (model values), based on the prior parameter vector $\hat{p}$, obtained after the previous estimation cycle. Then the deviation between the current model values $y_j=(y_{j1},...,y_{jn})$ and the prior model values $\hat{y_j}=(\hat{y}_{j1},...\hat{y}_{jn})$ is computed and multiplied by a weighting factor $w_{p,ij}$ for $i=1,...,n$ and $j=1,...,k$ for regulation of the prior’s impact. This term is also called ’forgetting term’ or ’forgetting penalty’. In this contribution the weighting factor is determined by $w_{p,ij} = 4/n$ while $n$ is the number of data points used for re-estimation. This means a higher weight of the forgetting term when having only a few data points for re-estimation, and less weight of the forgetting term as soon as more data points are considered. This is intended because generally, it is more difficult to obtain reliable parameter estimates when using only two or three data points for re-estimation than having four or more data points. The forgetting term helps to remember what has been already learned about the dynamic behavior of the same process, enabling a kind of ’memory’ (serving as correction or confirmation). Furthermore, a penalty term $\Delta p^T c_{\Delta p}$ for the change in parameters is added, where $\Delta p^T$ describes the euclidean distance between the current (estimated) *p* and the prior parameter vector $\hat{p}$ (calculated by the euclidean norm of the differences) and $c_{\Delta p}$ is a constant value to regulate the impact of the prior information. This penalty term fulfills a similar purpose as the forgetting term. It discourages parameter movements without sufficient improvement in the model predictions [13]. More details on this regularization technique can also be found in [1].

The resulting dynamic optimization problem is defined by minimizing the following objective function over the set of model parameters [13]:

2 $$\begin{aligned} J(p) = \sum _{j=1}^{k} \sum _{i=1}^{n} w_{m,ij} \cdot (y_{m,ij} - y_{ij}(p))^2 + w_{p,ij} \cdot (y_{m,ij} - \hat{y}_{ij})^2 + \Delta p^T c_{\Delta p}. \end{aligned}$$

with $\Delta p = \frac{\gamma }{n_p} \Vert \hat{p}^s - p^s \Vert _2 = \frac{\gamma }{n_p} \sqrt{\sum _{i=1}^{n_p} (\hat{p}_{i}^s - p_i^s)^2}$ for scaled parameter vectors $\hat{p}^s$ and $p^s$ of length *n*_*p*_ (i.e. components are scaled to the interval [0,1]) and constant tuning parameter $\gamma$. It should be noted that the squared-error MHE technique can also be found without the ’forgetting term’ in equation 2 [12].

### Investigated suspension cell culture process

In this contribution, the subject of the investigation is an industrial CHO cell culture process containing a seed train comprising five shake flask scales and three bioreactor scales as well as the production scale, whereby the focus lies on the bioreactor part of the seed train which is composed by bioreactor 1 (40 L), bioreactor 2 (320 L) and bioreactor 3 (2,160 L). From experimental data (offline measurements), taken once a day, time profiles for viable cell density $X_\mathrm{v}$, viability *Via*, concentrations of glucose $c_\mathrm{Glc}$, glutamine $c_\mathrm{Gln}$, lactate $c_\mathrm{Lac}$ and ammonia $c_\mathrm{Amm}$ have been used. In this work, data from 10 seed train cultivations from six campaigns with cultivation times between 72 and 96 h per scale (meaning 4–5 measurement time points per scale including the measurement at time 0 (inoculation)) were used for investigation. Additional data sets have been generated for modeling purposes and first parameter estimations. Therefore, 12 batch cultivations in 4 flask scales (3 cultivations each) having filling volumes of 40, 70, 300 and 1500 mL were provided. They cover cultivation time spans of 264 h (11 days) each, meaning that also the stationary and death phase were included. Details, e.g. about controlled process parameters, analytics and data cleansing can be taken from [15].

### Kinetic model and seed train prediction

The applied kinetic model, containing six mostly Monod-type algebraic equations (description of growth rate, death rate, substrate uptake and metabolite production kinetics) and 17 model parameters describing cell culture dynamics of total and viable cell density, $X_\mathrm{t}$ and $X_\mathrm{v}$, as well as concentrations of glucose $c_\mathrm{Glc}$ , glutamine $c_\mathrm{Gln}$, lactate $c_\mathrm{Lac}$ and ammonia $c_\mathrm{Amm}$, can be taken from [15].

Seed train prediction starts over three consecutive bioreactor scales, containing two passaging steps between them and is updated stepwise at each new sampling point. Evaluation of the prediction performance was realized calculating the relative prediction error in percent (= absolute deviation between predicted and afterwards added test data, divided by the test data). This criterion provides an intuitive assessment of the obtained prediction accuracy.

Since there are different overlapping factors having an influence on predicting several consecutive scales, (impact of estimated model parameters, e.g. maximum growth rate, and impact caused by possible deviations of the initial states at the beginning of each cultivation scale), the initial concentrations at the beginning of each cultivation scale are assumed to be known up to a certain experimental error. Otherwise, it would be unfeasible to make direct conclusions about the impact of re-estimated model parameters on prediction performance. And the goal of this study is to learn about optimal updating strategies concerning model parameters and to develop an adequate workflow for model updating.

## Results and discussion

The first goal of this study was to find out, how many data points (sampling points) are necessary for model parameter estimation to obtain predictions of the process future with high accuracy, using the widely applied weighted least squares estimation (WLSE) technique (see Sect. Impact of estimation horizon on prediction performance using weighted least squares estimation (WLSE)). In this context, it was also investigated if old data points can be ignored for parameter estimation. Second, it has been investigated to what extent analysis of the previous estimation steps concerning differences in model parameters can improve prediction performance of further seed trains of the same process (Sect. One parameter set for all scales vs. individual parameter sets per scale—Impact on prediction performance using WLSE). The third goal was to investigate if the presented moving horizon estimation technique, which contains an arrival cost term and a parameter penalty term (as explained in Sect. Objective function), is able to improve prediction performance (see Sect. Comparison: parameter estimation including prior knowledge—moving horizon estimation (MHE) vs. weighted least squares estimation (WLSE)). Finally, an iterative learning workflow is presented (see Sect. Developed iterative learning workflow), containing the recommended steps for dynamic parameter estimation, based on the findings of this study.

### Application to an industrial CHO cell culture process—a case study

As a case study, a seed train data set (batch data set), consisting of three consecutive bioreactor scales was divided into the past (past data points, e.g. time interval $t_1,...,t_3$) and the future (future data points, e.g. time interval $t_4,...,t_{12}$) of the current seed train. The estimation horizon covers part of the past (or the whole past) and is used to re-estimate model parameters (see Fig. 2). Each time new test data are added, the estimation horizon changes. Prediction performance of the remaining ’future’ (= prediction horizon) is evaluated through the relative prediction error in percent which is the discrepancy between measured (observed) data and their expected (modeled) values, relative to the measured value. It was calculated for all six variables but since viable cell density is the main variable of interest only the results for viable cell density $X_\mathrm{v}$ are presented in the figures of these sections.

The model parameter values which are taken as initial parameter values in this study (prior to the re-estimation process), come from parameter estimation using shake flask data of the same process. To prevent identifiability problems, which is a typical challenge when applying complex non-linear models containing several model parameters, cultivations at different shake flasks scales have been performed including lag phase, exponential phase, stationary phase and death phase. These values (estimated means and coefficients of variation) are published in [15]. According to the used seed train data sets in this work which do not include the stationary nor the death phase, some model parameters have been kept fixed while others have been re-estimated. Otherwise, the parameter estimation process would face identifiability problems, e.g. when trying to estimate the maximum death rate based on data that do not include the death phase. Free parameters are the maximum specific cell growth rate $\mu _\mathrm{max}$, the Monod kinetic constant for glucose uptake $k_\mathrm{Glc}$ and for glutamine uptake $k_\mathrm{Gln}$, the Monod kinetic constant for glucose $K_\mathrm{Glc}$ and for glutamine $K_\mathrm{Gln}$ as well as the kinetic production constants (yield coefficients) $Y_\mathrm{Lac/Glc}$ and $Y_\mathrm{Amm/Gln}$. Based on the coefficients of variation obtained from shake flask data, the boundaries for the parameters subject to re-estimation have been set to $\pm 50\%$ in this study.

#### Impact of estimation horizon on prediction performance using weighted least squares estimation (WLSE)

The following investigations deal with the question of how many data points should be used for re-estimation of model parameters (= model updating) using the weighted least squares estimation (WLSE). First, the left horizon bound is kept fixed meanwhile the right bound of the estimation horizon is growing after each iteration step.

Second, the estimation horizon length is kept fixed, i.e. adding a new data point means that the oldest data point of the estimation horizon is discarded (see Fig. 2a, top right). Three different fixed horizon lengths *n* were investigated, $n=2$, $n=3$ and $n=4$.

**Fig. 3:**
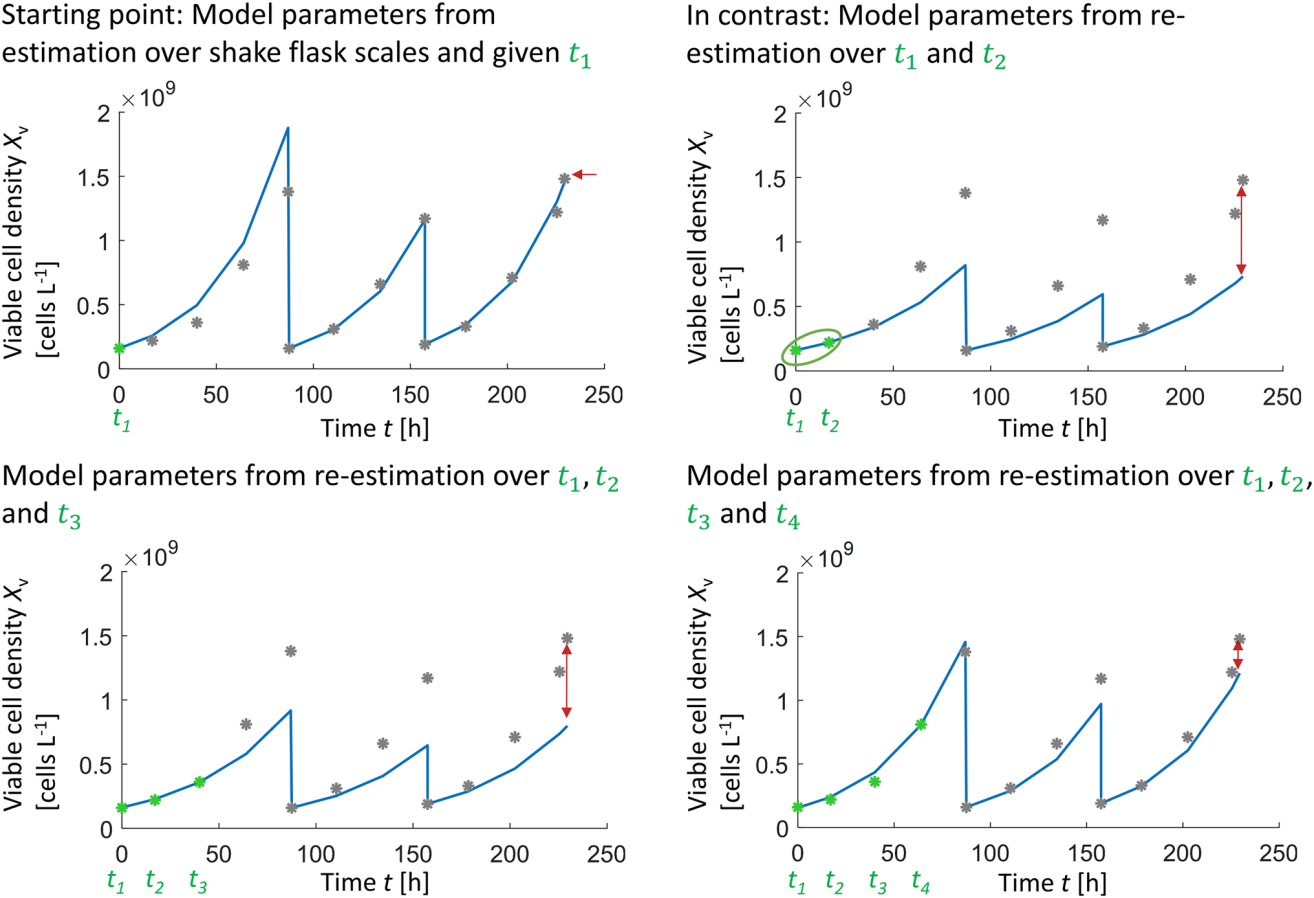
Viable cell concentration over time exemplary for seed train no. 1, before parameter estimation (top left), in contrast after re-estimation of model parameters based on data points at $t_{1}$ and $t_2$ (top right), after re-estimation of model parameters based on data points at $t_{1}$, $t_2$ and $t_3$ (bottom left) and after re-estimation of model parameters based on data points at $t_{1}$, $t_2$, $t_3$ and $t_4$ (bottom right)

Figure 3 shows exemplary a problem that can arise, if only two data points are used for model updating. The prediction top left is based on model parameters determined from flask scale experiments. As mentioned before, the initial concentrations of every scale are assumed to be known in this investigation in order to directly see the influence of model parameters on prediction performance. It becomes clear, that viable cell density of scale 2 and scale 3 can be predicted with high accuracy (5% and 4.2% relative prediction error). Taking measurement deviations of approximately 5% into account it can be concluded, that for this example the prediction error of scale 2 and scale 3 is more or less consistent (approximately at the same level) with the amount of irreducible uncertainty (within-lab precision). Prediction of scale 1 instead, shows a higher prediction error.

The seed train prediction in Fig. 3 top right grounds on model updating over two data points and it can be seen that prediction performance decreased for all three scales. If parameters change directly after model updating, two reasons could explain the decrease, a change in cell growth behavior or a change in parameters due to measurement errors. Measurement deviations could get too much weight if the estimation is performed only over two data points, because the estimation algorithm only focuses on minimizing the discrepancy between simulated and measured values at those two data points. Only, after addition of further data points it can be concluded which of both explanations may be appropriate. In the presented example, taking a third and fourth data point for model updating (bottom left and bottom right) indicates that the high prediction error after re-estimation over the first two data points (top right) probably grounds on measurement deviations.

**Fig. 4:**
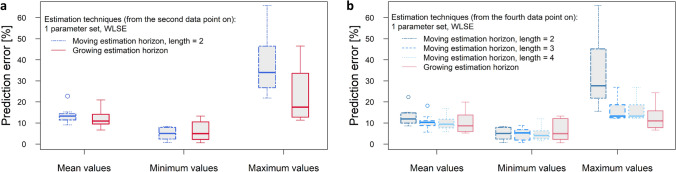
**a** Prediction error for viable cell density over 10 seed trains using weighted least squares estimation and 1 parameter set for all scales; moving estimation horizon of length 2 vs. growing estimation horizon (from the second data point on). **b** Prediction error for viable cell density over 10 seed trains using weighted least squares estimation and 1 parameter set for all scales; moving estimation horizon of length 2, 3 and 4 vs. growing estimation horizon (from the fourth data point on)

To compare the different estimation techniques more systematically, the mean of the relative prediction error over all re-estimation steps was calculated per seed train, as well as the minimum and the maximum prediction error. This was performed for 10 seed trains/batches and the results are presented through boxplots in Fig. 4a, b. The first group of boxplots within each diagram presents the mean prediction errors per batch over 10 seed trains, the second group shows the minimum values and the third group the maximum values.

In Fig. 4a two estimation techniques are compared over all re-estimation steps from the point in time $t_2$ on (meaning that at the first model updating step, concentrations at sampling points $t_1$ and $t_2$ are known and the concentrations within the prediction horizon = remaining future, $t_3,...t_\mathrm{end}$, are predicted). A moving estimation horizon with a fixed horizon length of 2 data points is compared to a growing estimation horizon (keeping the left bound fixed at $t_1$). It can be seen that taking only 2 data points for re-estimation of model parameters sometimes leads to low prediction accuracy (high maximum values for the relative error between 21 and 66%, left boxplot of maximum values). As mentioned earlier, this is not surprising because the measurements contain measurement deviations and those are getting too much weight if the estimation is performed only over two data points.

For comparison of larger estimation horizon lengths (containing more than 2 data points for re-estimation of model parameters), the relative prediction errors were considered from point in time $t_4$ on. The aim is to guarantee a fair comparison. When applying re-estimation over a horizon length of 4 data points, then the first considered prediction results are obtained for the prediction horizon $t_5,...,t_\mathrm{end}$, that means starting with $t_5$. No prediction on prediction horizon $t_3,...,t_\mathrm{end}$ can be made, neither for $t_2,...,t_\mathrm{end}$. Therefore, the comparison starts with the prediction horizon $t_5,...,t_\mathrm{end}$. The results are presented in Fig. 4b. Taking at least three data points for estimation typically reduces the undesired effect described for two data points (lower maximum values, second boxplot of group maximum values). Taking the four recent data points for parameter estimation lead to a similar prediction performance as when taking three data points (averaged batch mean over 10 batches: 12.9% relative prediction error for horizon length $=$ 2, 10.6% for horizon length $=$ 3 and 10.3% for horizon length $=$ 4). The best results are obtained if the whole past is used for model updating (9.99% averaged batch mean over 10 batches), especially concerning the maximum prediction error over one re-estimation cycle (fourth boxplot of group maximum values.

A detailed posterior analysis of the obtained model parameters at every updating step revealed further findings. If for example scale three should be predicted and model updating was performed including data from scale 1 and scale 2, the resulting prediction for scale 3 is not as good as a prediction based on shake flask experiments. This is due to slight differences in some model parameters for scale 1 compared to scales 2 and 3 (maximum growth rate $\mu _\mathrm{max}$ shows a difference of 7% on average). So, prediction for scale 3, based on model updating over scale 1 and scale 2 is a compromise between the behavior in both scales and therefore not optimal for scale 3. This observation is already mentioned in [15]. Obviously, the lower cell growth in reactor scale 1 occurs because mammalian cells have to adapt to different cultivation conditions (shaken system to stirred system). Variation of cell growth behavior is a common challenge in cell cultivation and reported among others in [43].

There may be differences in cell growth between different cultivation systems and also between different cultivation runs (seed trains). The latter can be taken into account through model parameter updating after adding new process data over a growing time horizon within a seed train. Differences in model parameters between cultivation scales could be detected as soon as one or more whole seed train data sets are collected. Then, model updating for further seed trains can be performed for each scale individually. In the next section, it was investigated, how model updating for each scale individually can change prediction accuracy for the remaining process future.

#### One parameter set for all scales vs. individual parameter sets per scale—Impact on prediction performance using WLSE

**Fig. 5:**
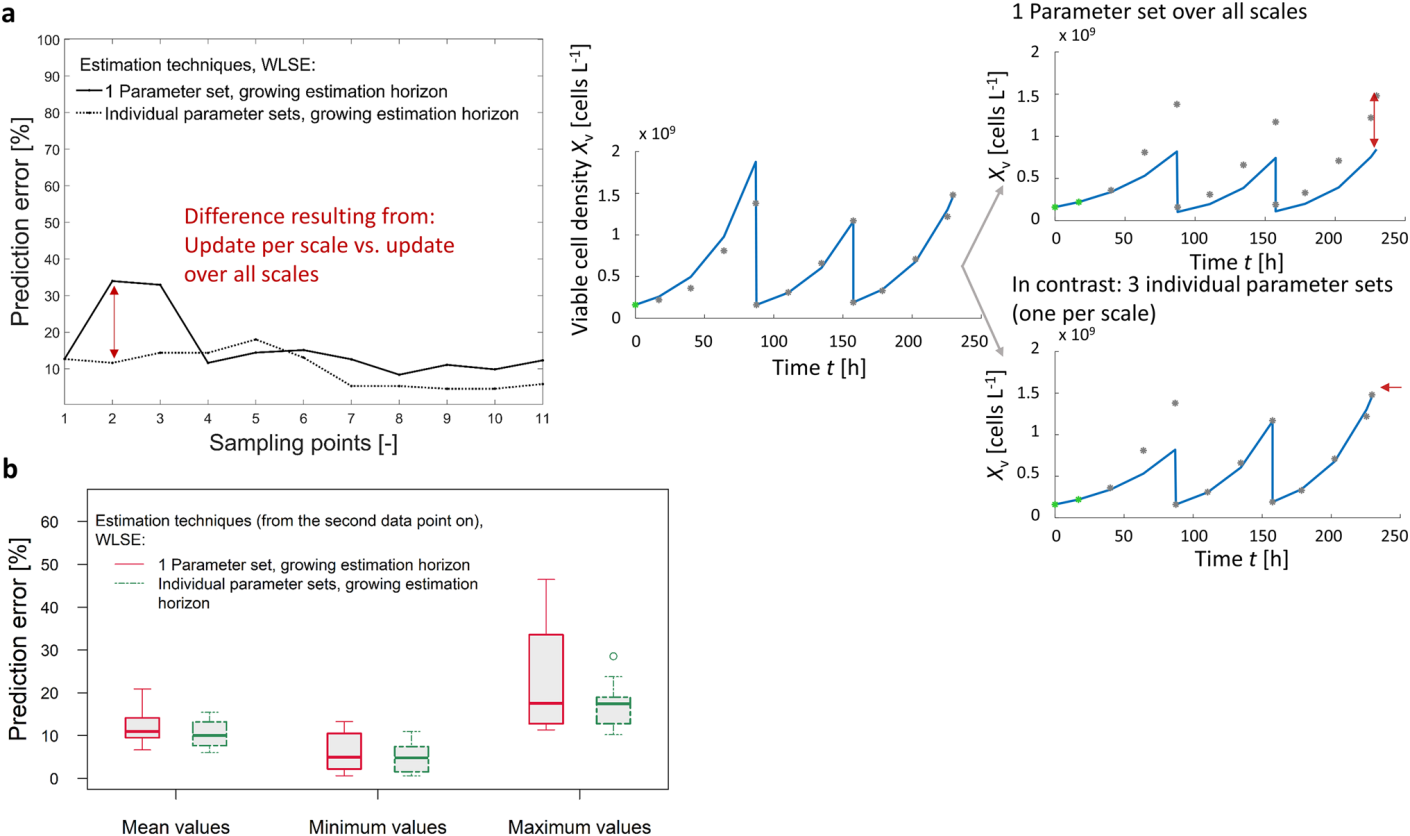
Comparison of prediction error for viable cell density using weighted least squares estimation; Re-estimation of one parameter set over all scales (growing estimation horizon) vs. three parameter sets (re-estimation of one parameter set per scale, over growing estimation horizon). **a** Exemplary for seed train no. 1, **b** results of 10 seed trains (from the second data point on)

Figure 5 shows the results for the relative prediction error if model parameters are re-estimated for each scale individually over a growing estimation horizon, keeping the model parameters for the other two reactor scales fixed. These results are compared to the previously described parameter estimation technique updating only one parameter set for all scales with a growing estimation horizon.

Figure 5a shows exemplary for one seed train how the relative prediction error develops within one sample-to-sample cycle. It becomes clear, that especially at the beginning of scale 1 (when only two or three data points are available), prediction performance of the remaining future can be improved by using individual parameter sets per scale.

Figure 5b shows the results over 10 seed trains. It can be seen that prediction performance improves in terms of mean and minimum values, but especially concerning the maximum prediction error values. Therefore, it is recommended to analyze the updated model parameter values concerning differences between scales, as soon as seed train data are collected. Nevertheless, relative prediction errors of nearly 20% (maximum values) are still not entirely satisfying and another estimation technique which takes more information concerning model parameters into account was investigated. When several seed train cultivations are performed, a lot of knowledge about growth behavior, which is expressed in form of corresponding model parameters, can be collected. Consequently, re-estimation of model parameters for the consecutive seed train does not have to start from the same starting point as the first seed train. A common way to induce knowledge concerning model parameters is to set corresponding starting values and boundaries for the optimization algorithm. Nevertheless, within the given ranges the optimization algorithm using a common WLSE approach only focuses on minimizing the discrepancy between data of the current estimation horizon and corresponding simulations. The MHE approach, in turn, embeds a sort of memory, helping the optimization algorithm ’not to forget’ which model parameter values are more plausible than others. A comparison of this approach to a common WLSE approach is presented in the following section.

#### Comparison: parameter estimation including prior knowledge—moving horizon estimation (MHE) vs. weighted least squares estimation (WLSE)

While, up to know, the sample-to-sample updating cycles were subject of investigation, now model updating is also investigated regarding the batch-to-batch updating cycles, meaning that knowledge learned within one sample-to-sample cycle is passed to the next sample-to-sample cycle. More specifically, this means the re-estimation of model parameters is performed using data of the current seed train (up to the current point in time) as well as information about model parameters from the last seed train. Technically, this can be realized using the moving horizon estimation technique described in Sect. Objective function. The objective function contains a term describing the discrepancy between the modeled values (e.g. for viable cell density) based on the prior model parameters (estimated during the previous seed train) and a penalty term for the deviation between prior and current model parameter values. The tuning parameter for this penalty term and the weighting factor for the prior model values were chosen as follows: $c_{\Delta p} = \frac{1}{n}$ and $w_p=\frac{4}{n}$, with *n*: number of data points within the estimation horizon. The dependence on *n* was introduced to reduce the influence of prior information when the database of the current seed train grows.

**Fig. 6:**
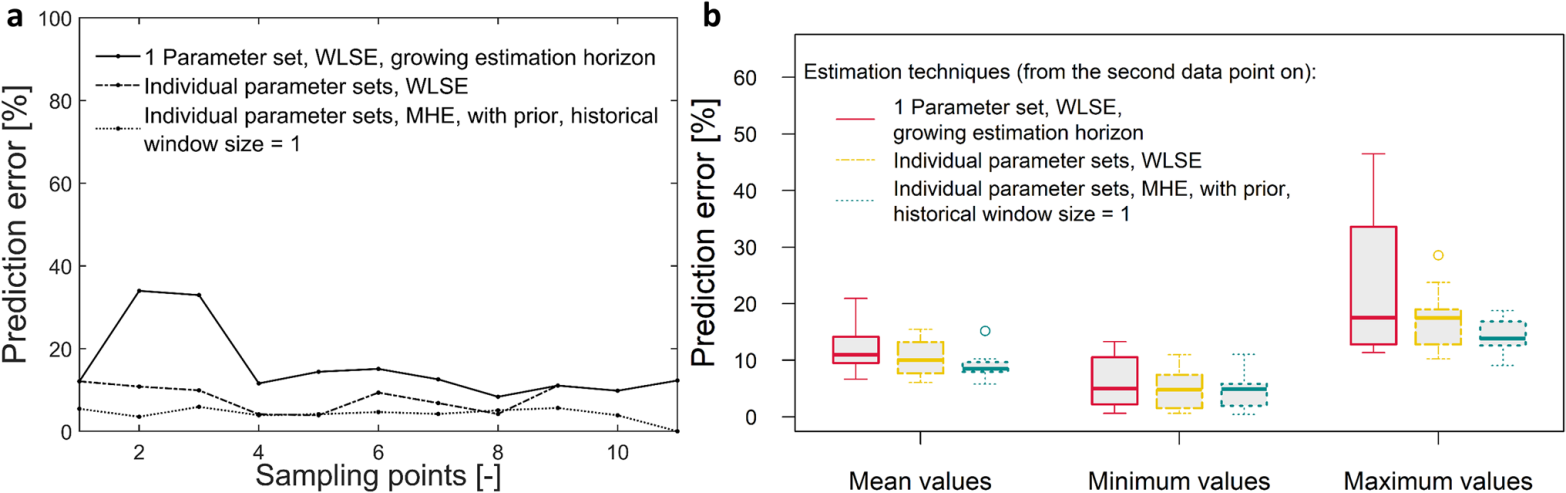
Comparison: Prediction error for viable cell density for three different re-estimation procedures. Re-estimation based on weighted least squares estimation (WLSE), without prior, i.e. which does not include any arrival cost term containing information from a-priori performed parameter estimation (re-estimation of one parameter set for all scales and re-estimation per individual scale). And as a third procedure, moving horizon estimation (MHE) is shown, which includes an arrival cost term containing information from a-priori performed parameter estimations (with prior), here from 1 seed train (arrival cost window = 1). **a** Prediction error (relative deviation between predicted and measured values) over sampling times exemplary for seed train no. 1; **b** Prediction error over 10 seed trains

Figure 6 shows the prediction results comparing the WLSE technique using 1 parameter set for all scales, using 1 individual parameter set per scale and the MHE technique using also 1 individual parameter set per scale and including prior knowledge. The diagram in Fig. 6 (left side) shows exemplary the development of the relative prediction error for one seed train and all three methods, showing that especially during the first model updating steps, the WLSE techniques, using only one parameter set for all scales shows a higher prediction error (> 30%) than using individual parameter sets (prediction error < 15%) or using the MHE approach (prediction error < 10%). An improvement can be achieved using individual parameter sets, one per batch. However, the best result is obtained using the MHE technique, even during the first updating steps when only very few data points are available for parameter estimation.

To obtain more representative results, the prediction error was evaluated over 10 seed trains. These results are presented in Fig. 6 (right side). It can be seen that the MHE technique leads to lower mean and maximum values of relative prediction error than the compared techniques (see Fig. 6 (right side) first three boxplots and last three boxplots). Moreover, it should be highlighted that there is a very low variation of the prediction error during a sample-to-sample updating cycle which stands for a robust estimator. In sect. Impact of estimation horizon on prediction performance using weighted least squares estimation (WLSE) it has been shown that estimation over two data points can lead to low prediction accuracy (relative prediction error sometimes higher than 60%) in the presence of measurement deviations. The MHE approach turned out not to be sensitive to measurement deviations or outliers. Prediction errors of more than 20% were not observed over 10 tested seed trains and the mean comes to 9%.

It has been shown that a possible bias caused by measurement deviations can be prevented this way. Furthermore, this benefit can be achieved without high computational costs, because learned information is included without increasing the database used for parameter estimation. Another benefit is, that batch-to-batch variabilities and variabilities between cultivation systems can be taken into account.

Furthermore, it was investigated if it is necessary to go 1, 2 or 3 seed trains back and to learn from these cultivations to receive an optimal prediction.

**Fig. 7:**
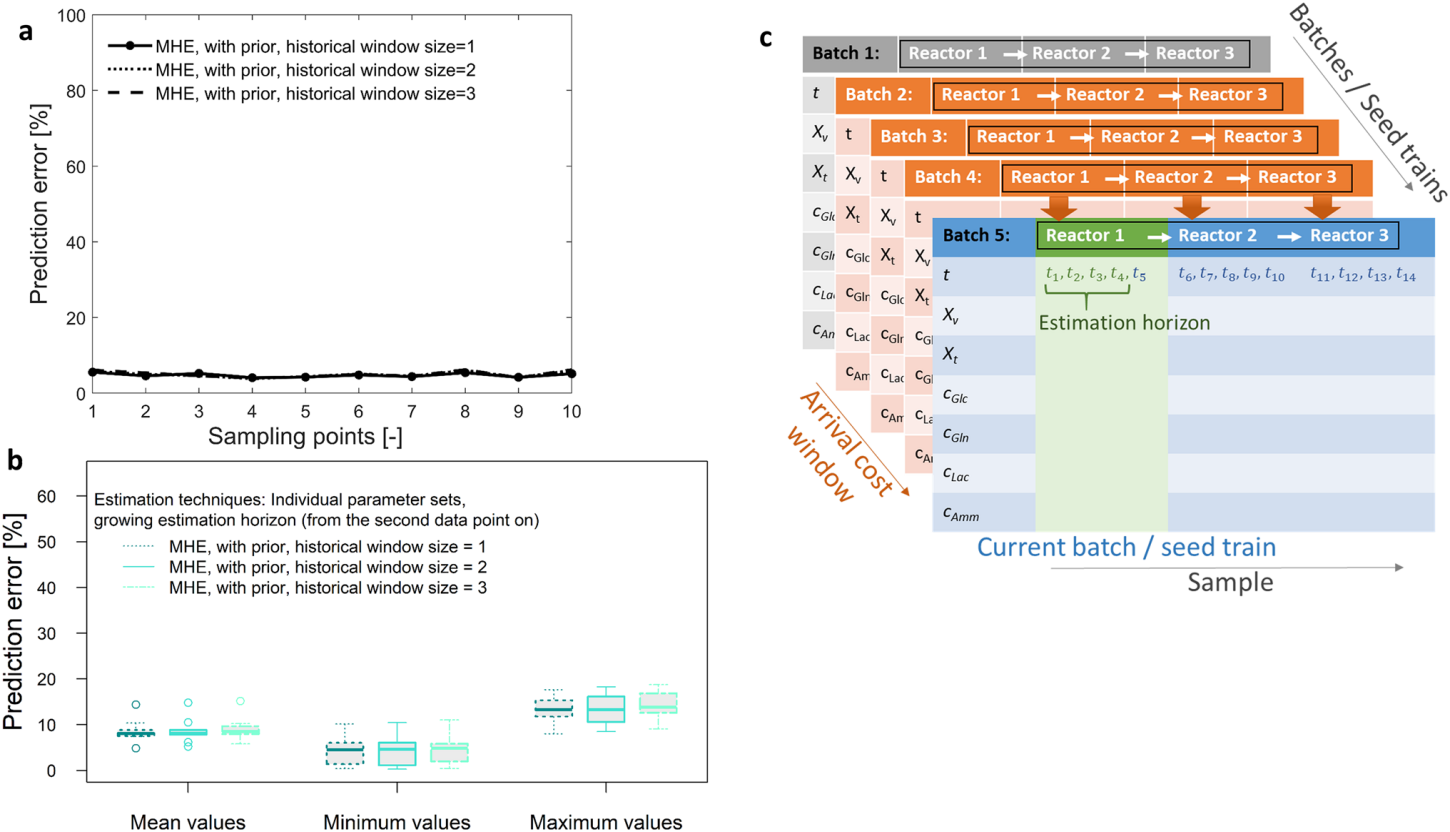
Impact of historical (arrival cost) window size for the MHE technique. **a** Prediction error for viable cell density at different sampling points and for a growing estimation horizon, exemplary for seed train no. 1, **b** prediction error for viable cell density over 10 seed trains for a growing estimation horizon (from the second data point on) and **c** the corresponding updating scheme

The following results show what happens if three consecutive batch-to-batch cycles are performed. This means that learned knowledge from seed train one is used for re-estimation and prediction of seed train 2. The obtained information is used for seed train 3 and the updated knowledge after seed train 3 is used for seed train 4 (see Fig. 7a). This could be also described as a arrival cost window size of 3 seed trains / batches. The results presented in Fig. 7 indicate that it is not necessary to include more than one historical seed train. The example in Fig. 7a show that there is hardly any difference in prediction performance between the three arrival cost window sizes and at all points in time, a relative prediction error less than 10 % has been achieved. Although, there are differences between the investigated seed trains, Fig. 7b shows that most seed trains show a mean prediction error of approximately 9 % and maximum values of approximately 14 %, not exceeding 18%.

### Developed iterative learning workflow

**Fig. 8:**
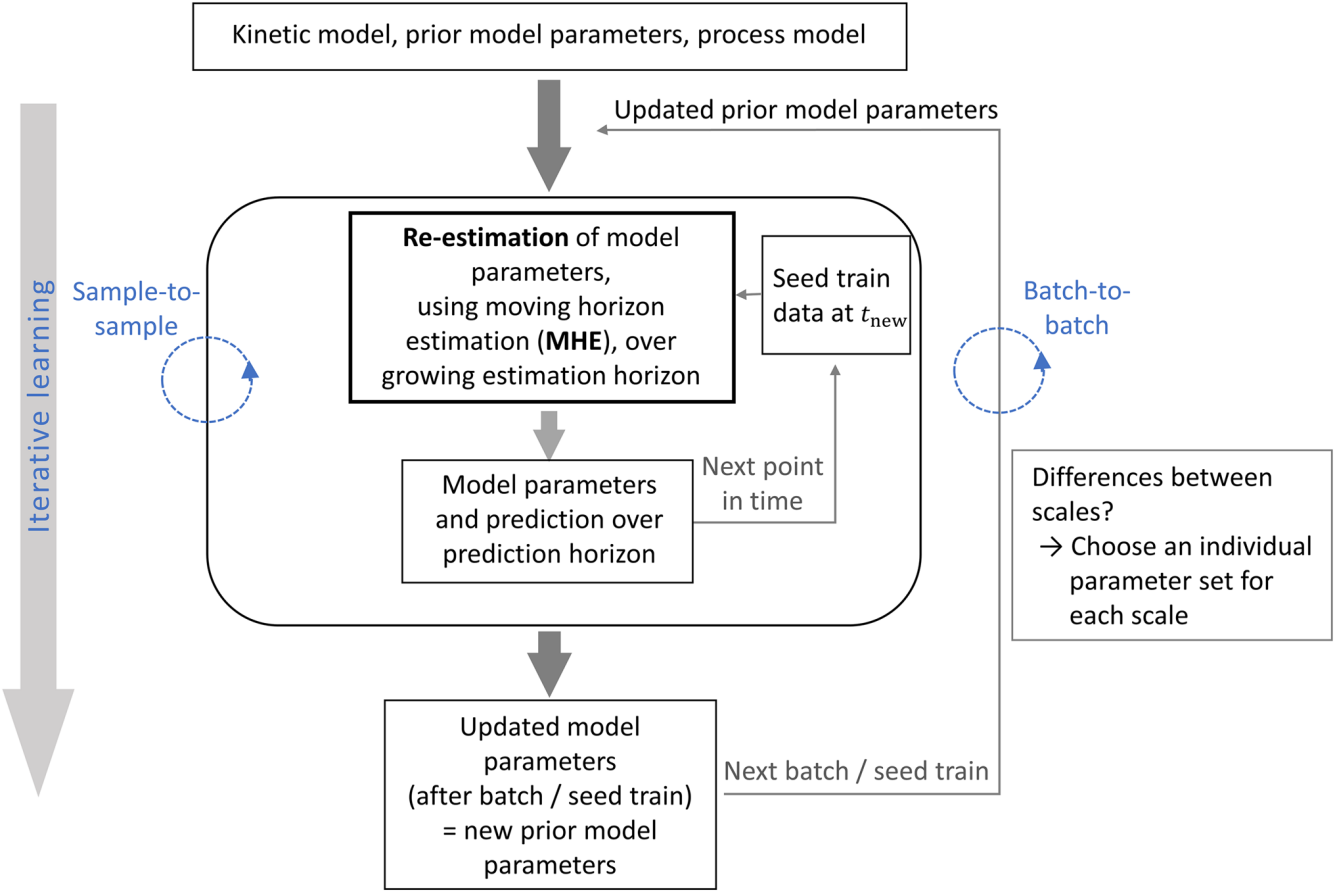
Proposed iterative learning workflow. Incorporation of knowledge (combining previous knowledge and new knowledge) during the whole process progress through sample-to-sample updating cycle during a batch/seed train and batch-to-batch updating cycle between batches/seed trains

Based on the findings obtained in this study, a workflow for seed train prediction was deduced illustrating the required steps to account for variabilities and possible differences between scales. The concept of the proposed workflow (see Fig. 8) is to iteratively include knowledge gained from ’new’/current data, without discarding the knowledge already obtained during the previous steps.

After providing a kinetic model, model parameters from previous experiments and a process model (e.g. containing equations for the computation of passaging between two scales/vessels) a new seed train for given initial concentrations is considered. Samples are taken in specific time steps and the following ’sample-to-sample’ updating cycle is performed. At every new sampling point, the model is updated, meaning that model parameters are re-estimated through the moving horizon estimation technique taking model parameters from previous experiments into account in form of additional terms within the objective function (see eq. 2).

The now obtained model parameters are used as the new prior model parameters in form of additional terms within the objective function for the next cultivation run and so on (batch-to-batch updating cycle).

Moreover, if differences between scales are observed it is advisable to use different model parameter sets for each individual scale and to update them individually (first model parameters for the first scale, then for the second scale and so on). Then the next cultivation run (e.g. the next seed train within the same production process) is considered. A new ’sample-to-sample’ updating cycle is performed, including learned knowledge from the first sample-to-sample updating cycle.

This proceeding leads to the results presented in Subsect. Comparison: parameter estimation including prior knowledge—moving horizon estimation (MHE) vs. weighted least squares estimation (WLSE). Compared to a common weighted least squares approach, prediction accuracy for viable cell density could be improved this way from 16.7 to 8.6% mean values and from 38.8 to 13.2% maximum values. Moreover, the standard deviation of the relative prediction error has been reduced from 9.5 to 2.9% relative prediction error. These results emphasize the robustness of the proposed estimation workflow.

### Common features of MHE and Bayesian parameter estimation

The estimation techniques applied in this study belong to the group of point estimates, meaning that for each future point in time within the prediction horizon, only one value per state variable is predicted (e.g. one value for viable cell density at a point in time $t_1$, one value at point in time $t_2$ and so on).

Meanwhile, the Bayesian approach belongs to the group of interval estimates, meaning that for every future point in time a whole interval (e.g. a 90%-interval) containing the possible values for a state variable is predicted, including the specific probability of occurrence for each value. In other words, prediction can be made at every future point in time including information about the predictive uncertainty (possible deviation). Common features of Bayesian parameter estimation and the presented MHE approach are, that both techniques include prior knowledge concerning the possible model parameter values as a kind of ’memory’, integrated in the parameter estimation process. Especially when only a few data are available and these data contain measurement errors, this ’memory’ helps not to propagate the change in model parameters caused by the measurement deviation onto the prediction of the future process.

Despite the added values of the Bayesian approach mentioned in [15], the MHE-based workflow presented in this contribution may be useful for many applications, where it is desired to save computational cost and to use a parameter estimation process which is simple to implement.

## Conclusion

Two different estimation techniques (weighted least squares estimation (WLSE) and moving horizon estimation (MHE), having different objective functions) were applied and compared as well as the database used for re-estimation of model parameters.

When using the WLSE technique at least 3 data points are necessary to reduce the maximum values of relative prediction error for viable cell density to less than 25% for the applied set up. Moreover, if differences between cultivation scales are observed, individual model parameter sets should be updated per scale (see row three of the overview in Table 1). Concerning the compared estimation techniques WLSE and MHE, it turned out that the optimal solution was obtained using the moving horizon estimation technique (MHE) and individual set of model parameters per scale in case of differences between scales. High prediction accuracy, represented through a relative prediction error of less than 15% maximum values was achieved from the beginning on as well as less than 10% on average. This estimation technique requires prior information (estimated model parameters based on previous cultivation data), whereby estimation results based on one preceding seed train turned out to be sufficient.

**Table 1:** Summary table containing the mean and maximum values of the relative prediction error (Rel. pred. error) and the precision-weighted prediction error (Precision-weighted pred. error) for different estimation techniques (WLSE/MHE), estimation horizon, arrival cost windows and number of parameter sets (Par.-sets), here 1 parameter set for all three scales vs. 3 parameter sets = one per scale)

Estimation technique				Mean values		Max values	
WLSE/MHE	Estimation horizon	Arrival cost window	Par.- sets	Rel. pred. error [%]	Precision- weighted pred. error [-]	Rel. pred. error [%]	Precision- weighted pred. error [-]
WLSE	Whole past	none	1	11.8	2.5	23.2	4.9
	of current						
	seed train						
WLSE	The last 2	none	1	13.7	2.9	38.8	8.3
	data points						
WLSE	Past of	none	3	10.6	2.3	18.9	4
	current scale						
MHE	Past of	Shake	3	9.0	1.9	14.2	3
	current scale	flasks					
MHE	Past of	1 previous	3	8.6	1.8	13.2	2.8
	current scale	seed train					
MHE	Past of	2 previous	3	8.6	1.8	13.5	2.9
	current scale	seed trains					
MHE	Past of	3 previous	3	9.0	1.9	14.7	3
	current scale	seed trains					

These results were then formulated in form of an iterative learning workflow which is presented in Sect. Developed iterative learning workflow. The performance of this workflow is described by the relative prediction error shown in row 6 of Table 1 (Arrival cost window: 1 previous seed train). A mean value of 8.6% relative prediction error and maximum values of 13.2% on average were achieved for viable cell density.

To properly asses these results concerning prediction error, the intermediate precision (within-lab reproducibility) of viable cell density should be taken into account. As stated in [15] it is expressed as $4.7\%$ coefficient of variation for the investigated process. Now, the prediction error has to be considered in relation to the intermediate precision, giving the precision-weighted prediction error. Values close to 1 mean, that the prediction error is more or less the amount of irreducible uncertainty which stands for stable models representing the stochastic nature of the environment. Therefore, low values are desired, meaning that there is not much reducible uncertainty. As can be seen in Table 1 columns 6 and 8, applying the MHE lead to a precision-weighted prediction error of 1.8–1.9 on average in comparison to 2.3–2.9 on average for the WLSE which shows the superiority of the MHE-based iterative learning workflow.

Advantages of this workflow are that process knowledge is generated and incorporated during the process and possible variabilities (e.g. batch-to-batch variabilities concerning maximum cell growth) can be taken into account which could lead to improved decision making (e.g. regarding points in time for passaging or split ratios). Problems arising from re-estimation over very few data points (e.g. bias due to measurement deviation when re-estimating over two sampling points) can be mitigated through the integration of prior knowledge within the objective function. Furthermore, the computational cost can be kept manageable because past data points from previous cultivation runs don not have to be used for re-estimation of model parameters. However, the learned knowledge from these cultivations flow in the re-estimation process. The general form of this framework allows the application to other bioprocesses as well as an implementation as part of predictive control methods.

## Abbreviations

MHE: Moving horizon estimation
WLSE: Weighted least squares estimation
OLFO: Open-Loop-Optimal-Control
CHO: Chinese hamster ovary

## List of symbols

$\Delta p^T$: Euclidean distance between *p* and $\hat{p}$ (-)
$\gamma$: Constant tuning parameter (-)
*μ*_max_: Maximum specific cell growth rate (h^−1^)
$c_{\Delta p}$: constant term for regulation of the prior information (-)
$c_\mathrm{Amm}$: Ammonia concentration (mmol L^−1^)
$c_\mathrm{Glc}$: Glucose concentration (mmol L^−1^)
$c_\mathrm{Gln}$: Glutamine concentration (mmol L^−1^)
$c_\mathrm{Lac}$: Lactate concentration (mmol L^−1^)
*i*: Running index (-)
*J*: Objective function (-)
*j*: Running index (-)
*k*: Number of variables (-)
*K*_S,Gln_: Monod kinetic constant for glutamine (mmol L^−1^)
*K*_S,Glc_: Monod kinetic constant for limiting substrate (mmol L^−1^)
*K*_Glc_: Monod kinetic constant for glucose uptake (mmol L^−1^)
*K*_Gln_: Monod kinetic constant for glutamine uptake (mmol L^−1^)
*n*: Length of estimation horizon (= number of measurement time points within the estimation horizon) (-)
*n*_*p*_: Number of parameters (-)
$p=(p_1,...,p_{n_p})$: Model parameter vector of length *n*_*p*_
$p^{s}$: Scaled model parameter vector
$\hat{p}$: Prior model parameter vector (from past estimations)
$\hat{p}^{s}$: Scaled prior model parameter vector (from past estimations)
*t*_*i*_: Point in time with index *i* (h)
*Via*: Viability (%)
*w*_*m, ij*_: Weighting factor (-) for state estimation, for variable with index *j*, at point in time with index *i*
*W*_*m*_: Weighting matrix for state estimation
*w*_*p, ij*_: Weighting factor (-) for prior values, for variable with index *j*, at point in time with index *i*
*X*_t_: Total cell density (cells L^−1^)
*X*_v_: Viable cell density (cells L^−1^)
*y*: Matrix containing all simulated values within the estimation horizon
*Y*_Amm/Gln_: Kinetic production constant for ammonia (mmol mmol^−1^)
$y_{j}=(y_{j1},...,y_{jn})$: Vector containing simulated values for variable with index *j*
*y*_*j, max*_: Maximum simulated value of variable with index *j*
$\hat{y}_{j}=(\hat{y}_{j1},...,\hat{y}_{jn})$: Vector containing prior values for variable with index *j* (= simulated values based on prior parameter vector $\hat{p}$)
*Y*_Lac/Glc_: Kinetic production constant for lactate (mmol mmol^−1^)
*y*_*m*_: Matrix containing all measured values within the estimation horizon
$y_{m,j}=(y_{m,j1},...,y_{m,jn})$: Vector containing measured values of variable with index *j*

## References

[1] Anane E, López C DC, Barz T, Sin G, Gernaey KV, Neubauer P, Cruz Bournazou MN (2019). Output uncertainty of dynamic growth models: effect of uncertain parameter estimates on model reliability. Biochem Eng J.

[2] Arora N, Biegler LT (2001) Redescending estimators for data reconciliation and parameter estimation. Comput Chem Eng 25(11–12):1585–1599. 10.1016/S0098-1354(01)00721-9. https://www.sciencedirect.com/science/article/pii/S0098135401007219

[3] de Andrade RR, Rivera EC, Atala DIP, Filho RM, Filho FM, Costa AC (2009). Study of kinetic parameters in a mechanistic model for bioethanol production through a screening technique and optimization. Bioprocess Biosyst Eng.

[4] Degasperi A, Fey D, Kholodenko BN (2017). Performance of objective functions and optimisation procedures for parameter estimation in system biology models. NPJ Syst Biol Appl.

[5] del Rio-Chanona EA, Zhang D, Vassiliadis VS (2016). Model-based real-time optimisation of a fed-batch cyanobacterial hydrogen production process using economic model predictive control strategy. Chem Eng Sci.

[6] Deppe S, Frahm B, Hass VC, Hernández Rodríguez T, Kuchemüller KB, Möller J, Pörtner R (2020). Estimation of process model parameters. Methods Mol Biol (Clifton, N.J.).

[7] Dochain D (2008) Bioprocess control. Control systems, robotics and manufacturing series. Wiley, London. https://onlinelibrary.wiley.com/doi/book/10.1002/9780470611128

[8] Frahm B (2014) Seed train optimization for cell culture. In: Pörtner R (ed) Animal cell biotechnology. Methods in biotechnology, vol 1104. Humana Press, Totowa, p 355–367. 10.1007/978-1-62703-733-4_22 10.1007/978-1-62703-733-4_22 24297426

[9] Frahm B, Lane P, Atzert H, Munack A, Hoffmann M, Hass VC, Pörtner R (2002). Adaptive, model-based control by the open-loop-feedback-optimal (olfo) controller for the effective fed-batch cultivation of hybridoma cells. Biotechnol Progress.

[10] Haseltine EL, Rawlings JB (2005). Critical evaluation of extended Kalman filtering and moving-horizon estimation. Ind Eng Chem Res.

[11] Hass VC, Lane P, Hoffmann M, Frahm B, Schwabe JO, Pörtner R, Munack A (2001). Model-based control of hybridoma cell cultures. IFAC Proc Vol.

[12] Hedengren JD, Eaton AN (2017). Overview of estimation methods for industrial dynamic systems. Optim Eng.

[13] Hedengren JD, Shishavan RA, Powell KM, Edgar TF (2014). Nonlinear modeling, estimation and predictive control in Apmonitor. Comput Chem Eng.

[14] Hernández Rodríguez T, Frahm B (2020). Design, optimization, and adaptive control of cell culture seed trains. Methods Mol Biol (Clifton, N.J.).

[15] Hernández Rodríguez T, Posch C, Schmutzhard J, Stettner J, Weihs C, Pörtner R, Frahm B (2019). Predicting industrial-scale cell culture seed trains-a Bayesian framework for model fitting and parameter estimation, dealing with uncertainty in measurements and model parameters, applied to a nonlinear kinetic cell culture model, using an mcmc method. Biotechnol Bioeng.

[16] Hernández Rodríguez T, Posch C, Pörtner R, Frahm B (2020). Dynamic parameter estimation and prediction over consecutive scales, based on moving horizon estimation: applied to an industrial cell culture seed train. Prepr Authorea.

[17] Jewaratnam J, Zhang J, Hussain A, Morris J (2012). Batch-to-batch iterative learning control using updated models based on a moving window of historical data. Proced Eng.

[18] Kern S, Platas-Barradas O, Pörtner R, Frahm B (2016). Model-based strategy for cell culture seed train layout verified at lab scale. Cytotechnology.

[19] Kroll P, Hofer A, Ulonska S, Kager J, Herwig C (2017). Model-based methods in the biopharmaceutical process lifecycle. Pharm Res.

[20] Kuchemüller KB, Pörtner R, Möller J (2020). Efficient optimization of process strategies with model-assisted design of experiments. Methods Mol Biol (Clifton, NJ).

[21] Le H, Kabbur S, Pollastrini L, Sun Z, Mills K, Johnson K, Karypis G, Hu WS (2012). Multivariate analysis of cell culture bioprocess data-lactate consumption as process indicator. J Biotechnol.

[22] Liu Y, Gunawan R (2017). Bioprocess optimization under uncertainty using ensemble modeling. J Biotechnol.

[23] Liu C, Gong Z, Shen B, Feng E (2013). Modelling and optimal control for a fed-batch fermentation process. Appl Math Model.

[24] Love J (2007). Process automation handbook.

[25] Manheim DC, Detwiler RL (2019). Accurate and reliable estimation of kinetic parameters for environmental engineering applications: a global, multi objective, bayesian optimization approach. MethodsX.

[26] Mears L, Stocks SM, Sin G, Gernaey KV (2017). A review of control strategies for manipulating the feed rate in fed-batch fermentation processes. J Biotechnol.

[27] Medeiros EM, Posada JA, Noorman H, Filho RM (2019). Dynamic modeling of syngas fermentation in a continuous stirred-tank reactor: multi-response parameter estimation and process optimization. Biotechnol Bioeng.

[28] Möller J, Kuchemüller KB, Steinmetz T, Koopmann KS, Pörtner R (2019). Model-assisted design of experiments as a concept for knowledge-based bioprocess development. Bioprocess Biosyst Eng.

[29] Möller J, Hernández Rodríguez T, Müller J, Arndt L, Kuchemüller KB, Frahm B, Eibl R, Eibl D, Pörtner R (2019). Model uncertainty-based evaluation of process strategies during scale-up of biopharmaceutical processes. Comput Chem Eng.

[30] Narayanan H, Luna MF, von Stosch M, Bournazou MNC, Polotti G, Morbidelli M, Butté A, Sokolov M (2019). Bioprocessing in the digital age–the role of process models. Biotechnol J.

[31] Narayanan H, Sokolov M, Butté A, Morbidelli M (2019). Decision tree-pls (dt-pls) algorithm for the development of process: Specific local prediction models. Biotechnol Progress.

[32] Narayanan H, Sokolov M, Morbidelli M, Butté A (2019). A new generation of predictive models: the added value of hybrid models for manufacturing processes of therapeutic proteins. Biotechnol Bioeng.

[33] Navarro MA, Salari A, Milescu M, Milescu LS (2018). Estimating kinetic mechanisms with prior knowledge ii: behavioral constraints and numerical tests. J Gen Physiol.

[34] Nelder JA, Mead R (1965). A simplex method for function minimization. Comput J.

[35] Paul K, Rajamanickam V, Herwig C (2019). Model-based optimization of temperature and ph shift to increase volumetric productivity of a Chinese hamster ovary fed-batch process. J Biosci Bioeng.

[36] Pörtner R, Platas Barradas O, Frahm B, Hass CV (2017) Advanced process and control strategies for bioreactors. In: Current developments in biotechnology and bioengineering. Bioprocesses, bioreactors and controls, p 463–493. 10.1016/B978-0-444-63663-8.00016-1

[37] Rao CV, Rawlings JB, Lee JH (2001). Constrained linear state estimation—a moving horizon approach. Automatica.

[38] Schenkendorf R, Gerogiorgis DI, Mansouri SS, Gernaey KV (2020). Model-based tools for pharmaceutical manufacturing processes. Processes.

[39] Sommeregger W, Sissolak B, Kandra K, von Stosch M, Mayer M, Striedner G (2017). Quality by control: towards model predictive control of mammalian cell culture bioprocesses. Biotechnol J.

[40] Teixeira AP, Alves C, Alves PM, Carrondo MJT, Oliveira R (2007). Hybrid elementary flux analysis/nonparametric modeling: application for bioprocess control. BMC Bioinf.

[41] Ungarala S (2009). Computing arrival cost parameters in moving horizon estimation using sampling based filters. J Process Control.

[42] Walsh G (2018). Biopharmaceutical benchmarks 2018. Nat Biotechnol.

[43] Xie X, Schenkendorf R (2019). Robust process design in pharmaceutical manufacturing under batch-to-batch variation. Processes.

[44] Xing Z, Bishop N, Leister K, Li ZJ (2010). Modeling kinetics of a large-scale fed-batch cho cell culture by markov chain monte carlo method. Biotechnol Progress.

[45] Zeugmann T, Poupart P, Kennedy J, Jin X, Han J, Saitta L, Sebag M, Peters J, Bagnell JA, Daelemans W, Webb GI, Ting KM, Shirabad JS, Fürnkranz J, Hüllermeier E, Matwin S, Sakakibara Y, Flener P, Schmid U, Procopiuc CM, Lachiche N, Sammut C, Webb GI (2011). Particle swarm optimization. Encyclopedia of machine learning.

